# 

**Published:** 1863-11

**Authors:** 


### CONTENTS.

### ORIGINAL CONTRIBUTIONS.

ART. XXX. The Organic Index of Human Longivity. By W. Byrd Powell, M.D., of Covington, Ky., ........ 544
ART. XXXI. Lecture Introductory to the Fifth Annual Course of Instruction in the Chicago Medical College, Medical Department of Lind University, Delivered Oct. 12, 1863, ........ 550
ART. XXXII. Improved Methods of Treatment in Deformities. By E. Andrews, A.D., M.D., Professor of Surgery in Chicago Medical College, ........ 564

### SELECTIONS.

Radical Cure of Congenital Inguinal Hernia. By D. W. Cheever, M.D.,.. 570
Report of Three Cases of Amaurosis produced by Tobacco. By J. C. Wordsworth, Esq., F.R.C.S., ........ 576
On Infantile Paralysis. By Holmes Coote, Esq., F.R.C.S., ........ 579
On the Treatment of Malarious Fever by the Subcutaneous Injection of Quinine. By W. J. Moore, L.R.C.P.Ed., ........ 583

### BOOK NOTICES.

The Pharmacopœia of the United States of America. Fourth Decennial Revision, ........ 585
A Manual of Instruction for Enlisting and Discharging Soldiers: with Special Reference to the Medical Examination of Recruits, and the Detection of Disqalifying and Feigned Diseases, ........ 586

### EDITORIAL.

Medical Societies in this City, ........ 586
Indications of Progress, ........ 587
Chicago Medical Society, ........ 588
Matter for Reflection.—Unnecessary Amputation of the Leg, ........ 589
Chair of Medical Jurisprudence, ........ 595
Ridgewood's Disinfecting Powder, ........ 595
Clinical Lecture.—Vaccination. By Graily Hewitt, M.D., ........ 597
Hydrophobia.—Electricity, ........ 602
Nævus.—Tartar Emetic, ........ 602
Thapsus Verbascum, ........ 603
Cholera.—Subcutaneous Injection of Morphia, ........ 603

